# Supplementary material for: Splice-Junction-Based Mapping of Alternative Isoforms in the Human Proteome
Source: Cell Rep. Author manuscript; Available in PMC 2020 Jan 15. (PMC6961840; doi:10.1016/j.celrep.2019.11.026)
Supplement: 2 [file NIHMS1546469-supplement-2.zip › DF1/PXD010154/Testis/Testis_44_ANKAR_AVGSICIGNLIASLAHSR.pdf]

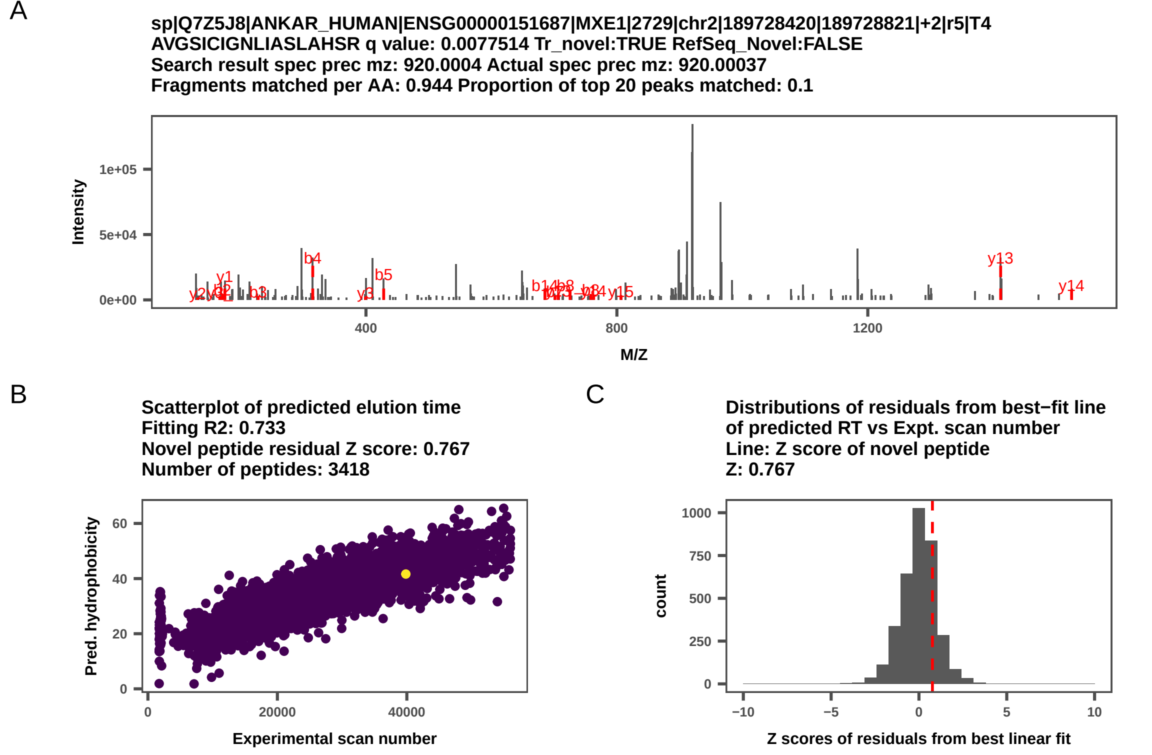
A
sp|Q7Z5J8|ANKAR_HUMAN|ENSG00000151687|MXE1|2729|chr2|189728420|189728821|+2|r5|T4
AVGSICIGNLIASLAHSR q value: 0.0077514 Tr_novel:TRUE RefSeq_Novel:FALSE
Search result spec prec mz: 920.0004 Actual spec prec mz: 920.00037
Fragments matched per AA: 0.944 Proportion of top 20 peaks matched: 0.1
Intensity
1e+05
5e+04
0e+00
b4
y1
b5
b3
y3
y13
y14
y15
M/Z
400
800
1200
B
Scatterplot of predicted elution time
Fitting R2: 0.733
Novel peptide residual Z score: 0.767
Number of peptides: 3418
Pred. hydrophobicity
60
40
20
0
0
20000
40000
Experimental scan number
C
Distributions of residuals from best−fit line
of predicted RT vs Expt. scan number
Line: Z score of novel peptide
Z: 0.767
count
1000
750
500
250
0
−10
−5
0
5
10
Z scores of residuals from best linear fit
